# A bimodal nomogram: a non-invasive tool to assist breast radiologists in decision-making

**DOI:** 10.1007/s00330-023-10357-0

**Published:** 2023-11-06

**Authors:** Marina Álvarez Benito

**Affiliations:** 1 Maimónides Biomedical Research Institute of Córdoba (IMIBIC), Córdoba, Spain; 2 Breast Cancer Unit, Department of Diagnostic Radiology, Reina Sofía University Hospital, Córdoba, Spain; 3 University of Córdoba, Córdoba, Spain

In recent decades, various imaging methods have been successfully incorporated into clinical practice for the study of breast cancer. However, mammography (MG) and ultrasound (US) continue to be by far the most widely used breast imaging methods.

MG is the accepted test for breast cancer screening, and every day a large number of mammograms are performed worldwide, with the aim of diagnosing a smaller breast cancer, improving the prognosis and therapeutic possibilities for this disease [1, 2]. Nonetheless, we know that mammography has limitations, the most important being low sensitivity, especially in women with dense breasts [3]. US is commonly used to supplement MG and improve the detection of mammographically occult cancers [4].

The Breast Imaging Reporting and Data System (BI-RADS) offers a standardized method to describe MG and US findings, assigns a category based on the probability for malignancy, and offers precise management recommendation [5]. It is not uncommon to find discrepancies between the resulting categories on MG and US. In these cases, the management option will depend on the highest category. If the result of one or both methods is a BI-RADS category 4 or 5, the recommendation will be to perform a biopsy [5]. Thus, although the combined use of MG and US improves cancer detection, it presents low specificity and a high number of benign biopsies. Approximately 57–71% of biopsies of lesions with discordant assessments are unnecessary, which increases healthcare costs and patient distress [6, 7]. It would be desirable to have the information provided by MG and US, maximizing cancer detection and minimizing the number of benign biopsies.

Nomograms are mathematical models that allow us to know the relationship between variables. Recently, nomograms have been used to predict individualized prognosis in cancer or to improve diagnosis in breast pathology [8–10]. In this issue of *European Radiology*, Xu et al aimed to develop a bimodal nomogram based on clinical, US, and MG imaging features and investigate its potential in improving cancer detection and reducing unnecessary biopsy when US and MG classifications are discordant [7]. This retrospective study was carried out with 706 women from two medical centers following opportunistic screening (614 patients) or diagnosis (92 patients) with discordant US and MG BI-RADS assessments, i.e., when one examination assessed a lesion as BI-RADS 4 or 5 and the other one assessed the same lesion as BI-RADS 0, 2, or 3. All cases were histologically confirmed (432 benign and 274 malignant) [7]. Univariate and multivariate logistics regression analyses were used to establish the independent risk factors of the nomogram. Age, MG imaging features (margin, shape, and density in masses, architectural distortion, and suspicious calcifications), and US imaging features (shape and margin in masses as well as calcifications) were independent predictors of breast cancer. All significantly independent factors were identified and then a bi-modal nomogram was constructed [7]. The nomogram obtained an area under the curve (AUC) of 0.87, 0.91, and 0.92 in the training, internal validation, and external testing samples, respectively, and demonstrated consistency in calibration curves [7].

The authors also presented the results of different imaging strategies: MG alone, US alone, MG + US, nomogram, MG + nomogram, and US + nomogram, comparing their diagnostic performances. Probably, the most important result of the study is that the nomogram presented better results than the use of US or MG alone, or the combination of MG + US. Additionally, coupling the nomogram with US or MG significantly improved the results, decreasing the number of unnecessary biopsies and missed cancers in comparison to US or MG alone [7]. The nomogram alone obtained a significantly higher AUC of 0.89, specificity of 0.86, and sensitivity of 0.80 than MG alone, US alone, and MG with US. The results of this study showed that the use of a simple modality (MG or US) is associated with a high number of missed cancers (90 with US and 124 with MG) [7]. The MG + US strategy obtained high sensitivity without missing breast cancer, but all women were biopsied in this strategy, and up to 61% of biopsies conducted were unnecessary. In contrast, coupling the nomogram with US reduced benign biopsies rate from 65 to 16% and the missed cancer rate from 13 to 2% compared to US alone. Similarly, coupling the nomogram with MG reduced benign biopsies rate from 49 to 13% and the missed cancer rate from 20 to 6% compared to MG alone [7].

Another interesting result of this study is that there was an increase in interobserver agreement between MG and US with the addition of the nomogram, with a weighted Kappa increasing from –0.708 (poor agreement) to 0.700 (substantial agreement), which enhanced the consistency of clinical decision-making [7].

The authors acknowledge that this nomogram is especially helpful in centers like theirs, where MG and US are performed by different radiologists. However, the nomogram can also be useful in centers where the two modalities are performed by the same radiologist as an adjunct tool to facilitate patient management.

Mass margins on US and MG and suspicious calcifications on MG were powerful indicators for predicting malignancy risk. Breast density may be another predictor for malignancy, but this variable was excluded in this study since the majority of patients had dense breasts. Furthermore, the nomogram, also took age into account, which provided a more individualized diagnosis. In this study, the risk of breast cancer was found to increase with age, and the authors suggest that close attention should be paid to older patients with discordant BI-RADS assessments [7].

Further improvement of nomogram is necessary because it still missed a certain amount of breast cancer (8%), compared to combined MG and US. More than half of missed cancers had an oval or round shape on US images [7]. Prospective studies with a large sample size and more external validation are needed to strengthen the robustness and generalization of the nomogram.

Nomograms that combine imaging findings and clinical data or radiomic analysis may further facilitate more accurate diagnoses and allow a personalized approach to breast pathology.

## Declarations

### Guarantor

The scientific guarantor of this publication is Marina Álvarez Benito.

### Statistics and biometry

No complex statistical methods were necessary.

### Study subjects or cohorts overlap

No.

### Methodology

• Commentary
